# The Mercury Behavior and Contamination in Soil Profiles in Mun River Basin, Northeast Thailand

**DOI:** 10.3390/ijerph16214131

**Published:** 2019-10-26

**Authors:** Rui Qu, Guilin Han, Man Liu, Xiaoqiang Li

**Affiliations:** Institute of Earth Sciences, China University of Geosciences (Beijing), Beijing 100083, China; qurui@cugb.edu.cn (R.Q.); lman@cugb.edu.cn (M.L.); xiaoqli@cugb.edu.cn (X.L.)

**Keywords:** mercury, soil contamination, spatial distribution, Mun River basin, northeast Thailand

## Abstract

To determine the geochemical characteristics and contamination of soil mercury in the Mun River basin, northeast Thailand, the vertical mercury distribution patterns and mercury contamination levels in six soil profiles under different land uses are studied. A total of 240 soil samples collected from agricultural land, abandoned agricultural land, and woodland were analyzed by an RA-915M mercury analyzer to determine the total mercury (THg) content, which ranged from 0.13 to 69.40 μg∙kg^−1^ in the study area. In the soil cultivation layer (0–30 cm), the average content of THg in the woodland (15.89 μg∙kg^−1^) and the agricultural land (13.48 μg∙kg^−1^) were higher than that in the abandoned agricultural land (4.08 μg∙kg^−1^), indicating that the plants or crops could increase the content of mercury in the surface soil layer. The total organic carbon (TOC) and iron content with high positive correlations with the THg content significantly contributed to the adsorption of soil mercury. Moreover, a higher pH value in the soil and a finer grain size in soil texture can be beneficial for the enrichment of mercury. A geoaccumulation index was used to evaluate the contamination of mercury, showing that this area had a slight contamination, and a few soil sites were moderate contamination.

## 1. Introduction

Mercury (Hg) is a global contaminant due to its high toxicity, bioaccumulation, and long residence time in the atmosphere during widespread transport, adversely affecting ecosystems and human beings [1,2,3,4]. Increasing mercury releases from human activities and increasing deposition of mercury to the soil [5,6,7] have resulted in severe soil pollution problems. Soil, as an essential medium for connecting atmosphere and water, plays a critical role in the global mercury cycle [8]. Soil is not only the sink of mercury but also the source of mercury, receiving the mercury input from the environment and re-emitting the deposit of mercury to the atmosphere [9], water [10] or plants [11]. Mercury contamination in the soil can suppress crops growth or kill plants, eventually affecting human health through bioaccumulation [12,13]. Therefore, soil mercury is closely related to the survival of humanity and the growth of crops [14]. As mercury pollutants have caught the interest of many researchers, some studies have already reported the behavior of mercury in the surficial environment around the world [15,16,17]. Thailand has undergone industrialization and urbanization development, as well as the increasing anthropogenic pollution deposited to the soil during this process [18]. However, these studies have been focused on mercury pollutants in the surface soil in Thailand except for the Mun River basin [19,20,21]. The lack of investigation into the vertical mercury distribution and content of mercury in the Mun River basin has made it difficult to identify potential contaminant problems. Moreover, a study on the controlling factors for mercury distribution behavior in different land use types is useful for providing basic ideas for solving potential mercury contamination in the future.

The objective of this study was to analyze mercury contamination, vertical mercury distribution patterns under different land uses, and the controlling factors of distribution to determine the geochemical characteristics of mercury in the Mun River basin, Thailand. Besides, the evaluation of mercury contamination can be used to provide advice for guiding agricultural activities in the future.

## 2. Materials and Methods

### 2.1. Study Area

The Mun River, located in the northeast of Thailand, is the main river system in the Korat Plateau. The Mun River, with a length of 673 km, covers 10 provinces and finally joins the Mekong river. The study area, the Mun River basin (14° N–16° N and 101°30′ E–105°30′ E), is the largest basin in Thailand with an area of approximately 82,000 km^2^ [22]. Within the basin, the terrain increases from east to west, and the area of the southwest is mountainous, while plains are distributed in the central and eastern regions [23]. The climate of the basin is humid subtropical [24], leading to the rainy and dry seasons. The annual precipitation is between 800 and 1800 mm [25]. The rainy season is from mid-May to mid-October and is affected by the southwest monsoon, with maxima rainfall generally in August or September. The dry season is from November to April and is generated by the northeast monsoon. The monthly average temperature is from 25 to 30 °C [25], and the highest temperature is in April. The fluvial deposit area where the Mun River flows through is primarily dominated by cretaceous claystone and sandstone with some halite and gypsum spread around the basin, while there is less than 10% coverage of tertiary basalt in the south of basin [22,26].

**Figure 1:**
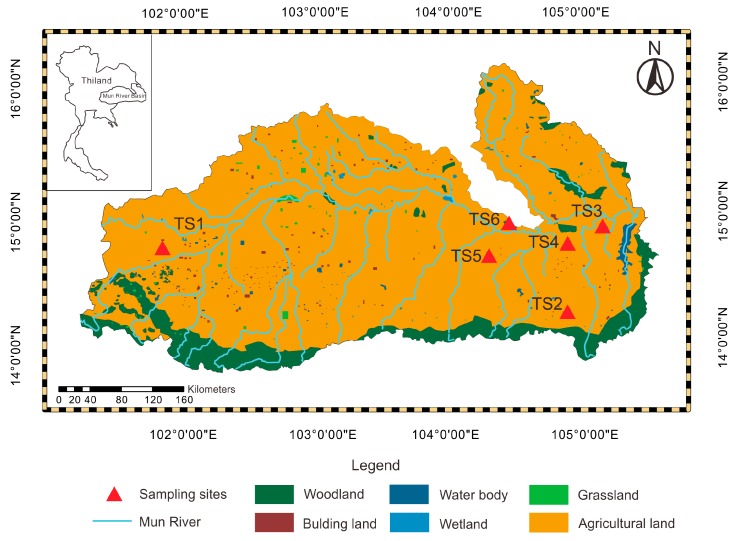
Land use types and locations of sampling sites in the study area.

As Thailand is a large rice-producing country, the main land use type is agricultural land in the Mun River basin, of which 75% is paddy land [25]. The primary rice cultivation technique is seeding, and it takes nearly a half year (July to November) for the rice to go from growth to ripe [27]. With the well-development of agriculture in the Mun River basin, a few industry areas are scattered in the basin. The dry land is main in the Upper Mun River, while the paddy land concentrates in Lower-Middle Mun River. The woodland and the grassland are mainly distributed in the south of the Mun River basin, and the rest of them are sporadically distributed around the basin (Figure 1). 

### 2.2. Sampling Collection and Sample Analysis Methods

Sample collection was implemented in March 2018. A total of 240 soil samples was collected from six profiles by taking different land use types into consideration, as shown in Figure 1. Additionally, the Thailand soil sites (TS) were chosen by considering intense agricultural activities, which are concentrated in the Upper Mun River and Lower Mun River. In order to compare the behavior and content of mercury in different land use types, the six sampling sites were set into agricultural land, abandoned agricultural land, and woodland. The location of each sampling site was recorded by a global positioning system (GPS), including the longitude and latitude coordinates; meanwhile, the land use type and soil conditions in the sampling sites were documented, as detailed in Table 1. In general, soil mercury content is affected by complicated factors from top to bottom; thus, a uniform sampling interval was performed in each sampling site to study the behavior of the soil mercury content. The different sampling intervals were based on the observation of the soil layer in the field, and a smaller interval was adopted in the complex soil conditions [28]. The color and particle of the soil in the TS5 profile change rapidly with soil depth. A 2 cm interval of sample collection was adopted in order to clearly show the variation of every property of the soil samples with depth. Sample collection was carried out by digging out each soil profile, using a tap measure to mark the depth, and then collecting the soil samples from bottom to top to avoid the mixed pollution. Every collected soil sample was about 2 kg.

The Hg (mercury) content was determined by an RA-915M mercury analyzer (Lumex Instrument, St. Petersburg, Russia) using the direct injection of a solid module, which was successfully applied in a previous study [29]. Compared to the atomic fluorescence spectrometer, this method directly focuses on soil samples without any digestion involved in the procedure; a lack of pre-treatment or digestion reduces the possibility of mercury loss and contamination during the process; meanwhile, this analysis is fast, accurate, and low cost. The standard reference materials GBW07402 (15 ± 3 μg∙kg^−1^) and GBW07405 (290 ± 30 μg∙kg^−1^) were provided by China National Standard Materials Research Center, and they were used to evaluate analyzer accuracy. The analyzer relative standard deviation (RSD) was 4.8%, and the detection limit was 0.10 μg∙kg^−1^ [29]. The Fe_2_O_3_ content was measured by ICP-OES (Optima 5300DV, Perkin Elmer, Waltham, MA, USA). The pH values were determined by a pH-meter (INESA Scientific Instrument Co., Ltd., Shanghai, China). The measurement of soil particle size was determined using a Mastersizer-2000 (Malvern Panalytical Ltd., Malvern, UK). The ISSS (International Society of Soil Science) method was used to describe the soil texture.

The mercury contamination of the study area was assessed by the geoaccumulation index (*Igeo*), the formula of which is:

(1) $$I_{geo}=Log_{2}\left[C_{n}/\left(k\times B_{n}\right)\right],$$

where *C_n_* is the concentration of the samples for metal n; *B_n_* is the background value of study area for metal n; and *k* (usually 1.5) is the correction index that is commonly used to characterize sedimentary characteristics, rock geology, and other effects. The mercury background value of soil in this study area was 11 μg∙kg^−1^ [20], which was from the ultisol soil type, which represented the majority of the agricultural soils in Thailand, meaning the *B_n_* was 11 μg∙kg^−1^. In general, the contamination level of the geoaccumulation index includes seven grades, and the risk of each level is shown in the Table 2.

### 2.3. Statistical Analysis

The statistical analysis was analyzed by SPSS 18.0 (SPSS Inc., Chicago, IL, USA). The relationships among the mercury content, the total organic carbon (TOC) content, the Fe content, and pH value were determined by regression analyses. The bivariate correlations with a Pearson correlation coefficient and two-side test in significance test were adopted; the coefficient *R*^2^ and *p*-values were determined while the fitting lines were drawn. The figures were drawn using Origin 9.0 (OriginLab Corporation, Northampton, MA, USA).

## 3. Results and Discussion

### 3.1. Content of THg in Soil

**Figure 2:**
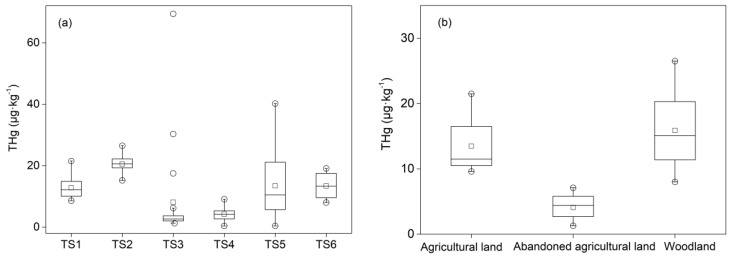
(**a**) The content of THg in each profile and (**b**) the content of THg in different land use types (0–30 cm) (o represents outliers, and □ represents mean values).

The total mercury (THg) contents in different sampling sites are shown in Table 3. In the TS4 and TS5 sampling sites, the content of mercury in the soil was not detected after 116 and 290 cm, respectively. Moreover, a sample in TS4 where the content of mercury was much higher than those in other profiles is shown in Figure 2a. 

In general, the average THg content tested in this study was 12.16 μg∙kg^−1^, ranging from 0.13 to 69.4 μg∙kg^−1^. The mercury content of the agricultural land topsoil (0–20 cm) in this study (9.8–21.5 μg∙kg^−1^) was higher than that of other paddy land (3.2–4.05 μg∙kg^−1^) [21] in Thailand. The average contents of THg were generally lower than those in different contamination sources have been reported by other researchers in Thailand, such as municipal waste in Samui Island (76–215 μg∙kg^−1^) [30] and mining in Phichit Province (16–181 μg∙kg^−1^) [31], indicating that the contamination from agriculture is lower than those from mining or municipal waste. 

Figure 2b shows distinct differences of the THg content among different land use types. In the depth of 0–30 cm, the average content of THg in the agricultural land and the woodland was 13.48 and 15.89 μg∙kg^−1^, respectively, while the average content in the abandoned agricultural land was 4.08 μg∙kg^−1^. A likely explanation for this is that the higher content of THg in the agricultural land and the woodland is a result of crops or plants, implying that the widespread contamination of mercury in the soil happens via agricultural activities [20]. The influence of using fertilizer, dung, and sludge during agricultural activities may result in the enrichment of mercury in the crop lands [32].

### 3.2. Vertical Distribution of THg in Soil Profiles

**Figure 3:**
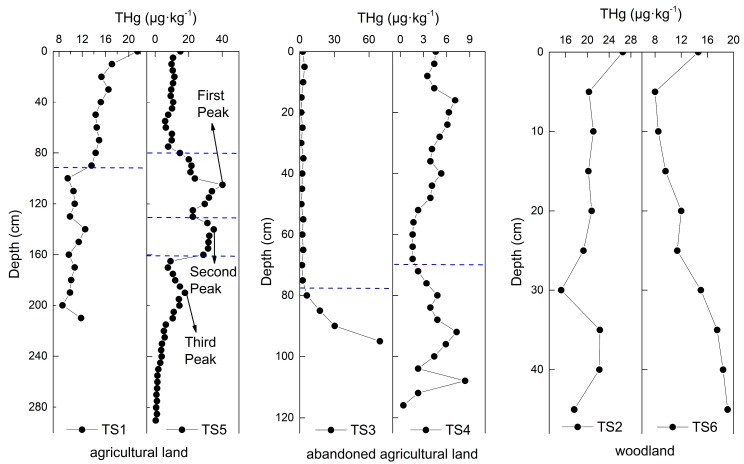
The vertical distribution of the total mercury in soil.

The vertical distribution of THg, divided into three types of land use: agricultural land, abandoned agricultural land, and woodland, as shown in Figure 3. In the agricultural land and the woodland, the content of THg in the surface soil (<2 cm) was a little higher than those at a depth of 2–30 cm. Liu et al. [33] observed the same phenomenon; a possible reason for this is that the surface soil covered by plants has not been not to be exposed to solar radiation, which makes the lessens the loss of mercury in the surface soil by solar reduction.

In the agricultural land, both TS1 and TS5 showed a homogenous distribution of THg in the surface soil and the deeper layer (<90 cm). However, in the medium and bottom soil, the content of THg in TS1 slightly decreased but was still homogenous, while the THg content in TS5 showed three peaks and was divided into three parts (80–130, 135–160, and 165–290 cm), implying that there were three controlling factors leading to these behaviors. The different vertical behaviors of TS1 and TS5 was due to the complicated variation in the soil of the TS5 profile. As shown in Table 1, compared with the lack of variation of soil found in the TS1 profile, several different types of soil and the occurrence of Fe-Mn nodules, which have a great adsorption of soil mercury [34], were observed in the TS5 profile, resulting in more complicated mercury distribution behavior. In the abandoned the agricultural land, there was a gradual increase of the THg content in the deep soil because of the occurrence of Fe-Mn nodules, which can absorb mercury in soil. Compared with the content of THg in the woodland soil, the TS2 profile showed a more homogenous distribution than the TS6 profile. The soil mercury increased with the depth in the TS6 profile, possibly due to the low content of the TOC, oxidizing conditions, acid pH value [35,36] and soil texture. 

### 3.3. Influence of Controlling Factors on the Distribution of THg

#### 3.3.1. Organic Carbon and Iron Oxides

**Figure 4:**
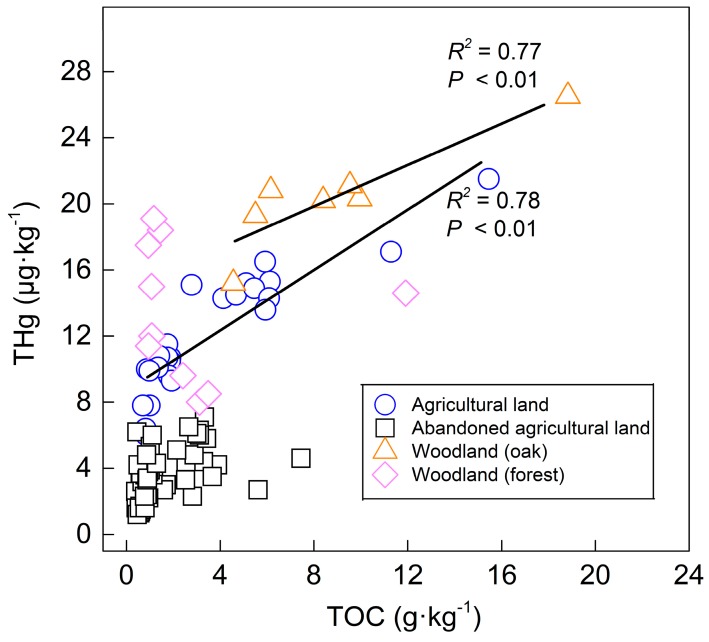
The relationships between the total organic carbon (TOC) and THg in different land use types (<90 cm).

With the objective of finding the main controlling factor on the distribution behavior of mercury in the soil profiles, the correlations between organic carbon, iron content, and mercury content were evaluated in this study. Organic carbon plays an essential role in enhancing the capacity of binding metals for soil [37] and affects the distribution behavior of mercury in soil [38]. Similar research findings have been reported to verify this correlation between the TOC and mercury [36,39,40], and a high TOC content can increase the baseline content of mercury [41], which was observed in this study area. As shown in Figure 4, the THg content of the surface soil and the deeper layer (<90 cm) in the agricultural land and the woodland (oak) showed significantly positive correlations with the TOC, indicating that a high content of the TOC is beneficial for the enrichment of mercury. The high affinity of mercury for the TOC is the reason for the higher THg content in the agricultural land and the woodland than that in the abandoned agricultural land. 

In comparison with the TS2 profile, the TS6 profile was supposed to have a high content of the TOC in the soil that was covered by thick leaves, but the result was not expected (Table 4). The high THg concentration with a low content of the TOC in forest soil showed no correlation between the TOC and THg in Figure 4. A possible explanation for this is that organic carbon is important in bioavailability [42], and the organic carbon can be decomposed by soil microorganisms. Wu et al. [43] stated that the light fraction of organic carbon is easily covered under forest soil and is readily decomposed by soil microorganisms. Pant and Allen [40] also reported that the decomposed light fraction of organic carbon might affect the Hg/TOC ratio, leading to a low coefficient of correlation between THg and the TOC under forest cover.

**Figure 5:**
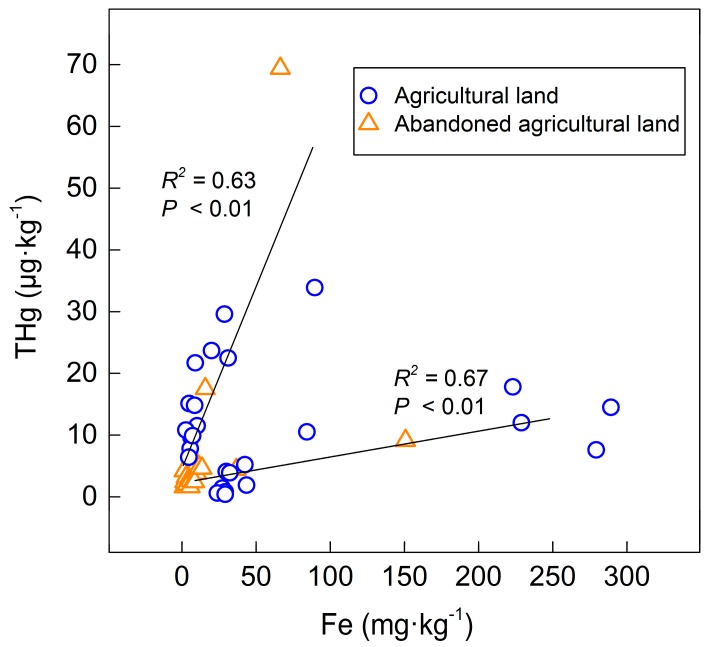
The relationships between the Fe content and THg in the agricultural land.

It has been reported in some studies that THg concentration decreases with increasing depth studies [15,17]. However, THg concentration increased in deep soil such as the abandoned agricultural land (TS3, TS4) and the agricultural land (TS5), as shown in Figure 3, where we found some iron rust and Fe-Mn nodules in the soil (Table 1). The profiles with Fe-Mn nodule presentation had a distinct variation of Fe content between the surface soil and deep soil, showing a large range of the Fe content and a high standard deviation (Table 4). As shown in Figure 5, both the agricultural land and the abandoned agricultural land showed similar correlation trends between the Fe content and the THg content. The different correlation trends in the same land use type indicate that the amount of mercury in the soil affects the adsorption efficiency of iron to mercury. 

Zarcinas et al. [20] adopted a principal component analysis of heavy metal in soil around Thailand and suggested that the content of mercury had a strong correlation with the Fe content. In this study, the Fe content in the deep soil was higher than that in the surface soil, showing that the mercury content increased in the deep soil of the abandoned agricultural land and the occurrence of first and third peaks of the agricultural land (TS5), as shown in Figure 3. 

#### 3.3.2. pH and Soil Texture

Soil properties are supposed to affect the behavior and content of metals [45]. The pH value in soil can strongly change the mobility of mercury by influencing the different presence forms of mercury. As pH increases in soil, the bioavailability and mobility of mercury decreases [46,47,48], and the content of mercury increases, which is in agreement with our results. In the agricultural land, the occurrence of mercury content decreased in TS1, and the second peak in TS5 (Figure 4) was attributed to the influence of pH value change. Though no significant variance of the TOC concentration occurred in TS1, the average content of mercury slightly decreased from 15.72 to 10.44 μg∙kg^−1^. This discrepancy could be attributed to the decrease in pH value, which reduced from 6.55 to 6.14. A decrease in pH may reduce the adsorption of mercury by soil or the TOC, or it may increase the mobility and bioavailability of mercury [49,50].

**Figure 6:**
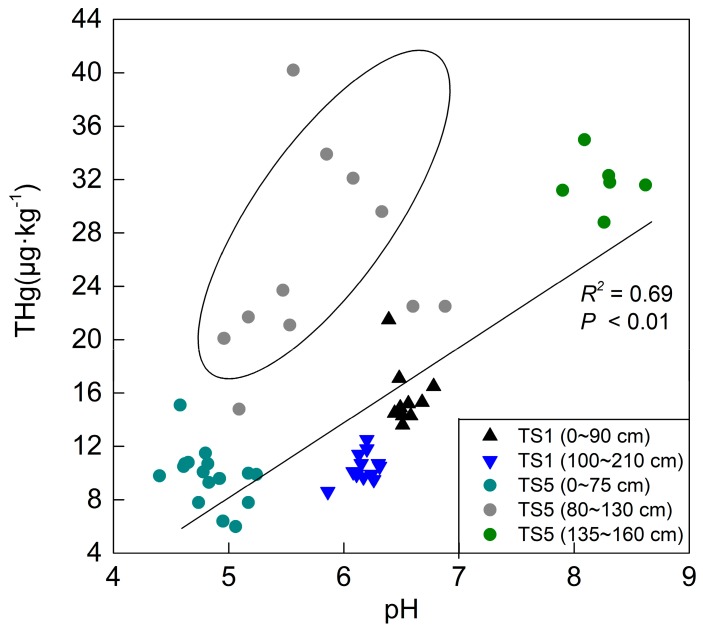
The correlation between pH and THg in the agricultural land.

Moreover, a similar explanation may be adopted in the vertical distribution of mercury in TS5. As can be seen in Figure 6, the points out of the circle are more concentrated and have a positive correlation trend, while the gray points in the circle are more disordered and were affected by adsorption of iron-containing minerals—the main controlling factor. The behavior of the gray points implies that using pH value to analyze mercury distribution behaviors does not rule out the influence of other factors, such as the TOC, iron-containing minerals, and soil texture. As mentioned above, the occurrence of the first peak (TS5; 80–130 cm) and third peak (TS5; 165–290 cm) (Figure 4) was attributed to the Fe content increase, which showed a strong positive correlation. However, there was no correlation between the Fe content and the THg content, and there were no disordered points (Figure 6) for the occurrence of the second peak (TS5; 135–160 cm), indicating that the increase of the mercury content could not be attributed to the iron content. The THg content increased with the increasing pH value in the agricultural land, as shown in Figure 6, and this result was applied to the explanation of the occurrence of the second peak in the TS5 profile.

With respect to soil texture, grain size was taken into account for the controlling factor of mercury distribution. Palmieri et al. [36] reported that clay, silt, and sand have similar distribution behaviors to mercury, suggesting that grain size primarily affects the content of mercury in soil. Liao et al. [51] stated that clay has the highest capacity of adsorption for mercury compared to loam and sand, implying the a smaller grain size is beneficial for the enrichment of mercury. The negative correlation between the content of THg and mean grain size was also reported by Zhao et al. [52]. In our study area, the results showed that the soil texture is mainly loam (Table 5), which is in agreement with another study [20]. The TS4 and TS5 profiles had different types of soil texture, while the other profiles had the same soil texture. In this study, the land use type showed no clear the relationship with soil texture. However, the soil texture of the abandoned land with less mercury had more sand content compared with the other profiles. The high sand content in the forest profile may provide an explanation for the increase of mercury with increasing depth, because sand is beneficial for enhancing the vertical mobility of mercury.

**Figure 7:**
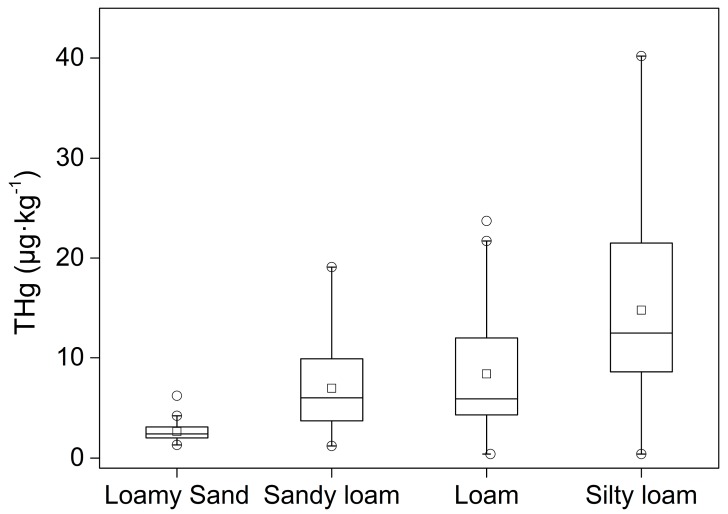
The THg content in different types of soil texture (o represents outliers, and □ represents mean values).

The total mercury contents were combined in all profiles according to the different types of soil texture (Figure 7). The soil texture was arranged from coarse to fine according to average grain size: loamy sand > sandy loam > loam > silty loam; this means that the fine soil texture had a lesser sand content. As shown in Figure 7, the finer grain size has a larger concentration range and a higher average value of total mercury in the soil, suggesting that the content of mercury is relative to soil grain size. As for profiles with different types of soil texture, like TS4 and TS5, although the distribution behaviors are dominated by the TOC, iron-containing minerals, and pH value, we could distinctly observe that the change of soil texture led to a change of the THg content (Figure 3).

### 3.4. Geoaccumulation Index

**Figure 8:**
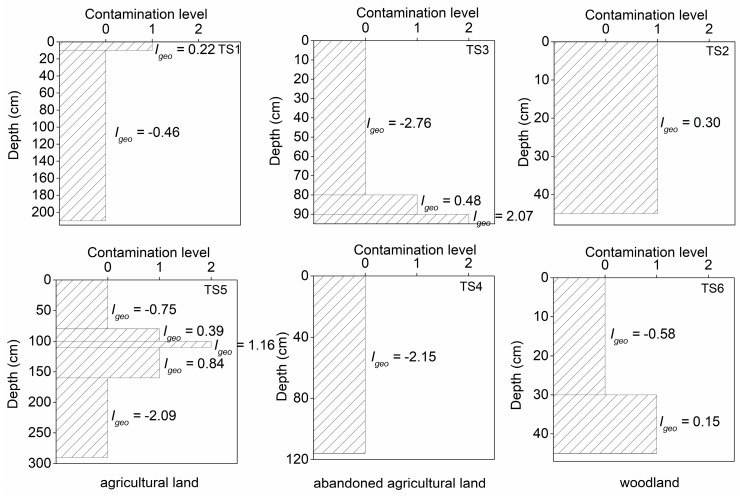
The mercury contamination level in the soil profiles of different land uses.

The mercury contamination level in the soil was evaluated by the geoaccumulation index (*Igeo*) in the Mun River basin. The ratio of mercury contamination level and graphs of contamination levels in different types of land use which changed with the depth are shown in Table 6 and Figure 8, respectively. In general, the range of contamination level in this study area was 0–2, which corresponds to uncontaminated to moderately contaminated levels. The soil samples in this study area were mainly slightly contaminated, and a small number of soil samples were moderately contaminated. All the soil profiles were contaminated with different contaminated levels except for TS4 (Table 6).

As shown in Figure 8, the mercury contamination was concentrated on the shallow and deeper soil (<100 cm). The moderate contamination occurred in the deeper soil of the profiles in the abandoned agricultural land (TS3) and the agricultural land (TS5), while the other situations were slightly contaminated. The contamination of the woodland was probably due to the agricultural land near the sites. Agricultural activities may have contributed to the mercury contamination of the soil, and the vertical mobility of mercury was likely beneficial for the enrichment in the deeper soil.

## 4. Conclusions

This study reported the content of total mercury, the characteristics of vertical distribution, and the controlling factors of distribution behaviors in the soil around the Mun River basin, Thailand. The THg concentration in the study area ranged from the 0.13 to 69.4 μg∙kg^−1^. The mercury content was higher in the agricultural land and the woodland than that in the abandoned agricultural land, suggesting that crops or plants are beneficial for the enrichment of mercury. The vertical mercury distribution behaviors in the Mun River basin soil were dominated by the TOC, iron-containing mineral, pH value, and soil texture. The distribution of mercury was homogenous in the surface soil and the deeper layer (<90 cm) within the agricultural land and the woodland, and it was mainly controlled by the TOC content, which had a strong positive correlation with the mercury content. Moreover, an explanation for the increase of mercury in the bottom soil is the adsorption of iron-containing minerals for mercury in the agricultural land and the abandoned agricultural land (TS3, TS4, and TS5), which showed a high correlation between the Fe content and the THg content. The ability of iron adsorption for mercury was based on the amount of mercury. Additionally, the pH value and soil texture made some contributions to the mercury distribution behavior. The increase of the pH value in the agricultural land resulted in the increase of the THg content. In our case, the mercury content, in sequence from high to low in different soil textures, was loamy sand, sandy loam, loam, and silty loam; this indicates that the finer grain size may have had a higher THg content. Moreover, the variation of the vertical mercury distribution may have been attributed to the change of soil texture in the profiles. Taken together, the geochemical characteristics of mercury were controlled by the TOC content, iron-containing minerals, pH value, and soil texture, which together determine the vertical distribution patterns of mercury.

Most sample sites (TS1, TS2, TS4, and TS6) are slight mercury contamination and a few sample soils (TS3 and TS5) are moderate mercury contamination. The mercury contamination was concentrated in the shallow and deeper layers (<100 cm) of the soil profiles, and the moderate contamination occurred in the deeper layer of the agricultural land and the abandoned agricultural land. Agricultural activities may lead to mercury contamination and contaminate the surrounding woodland. According to the pollution assessment results, in order to prevent mercury pollution and expansion, agricultural behaviors need to be monitored and managed. Agricultural behavior such as straw returning or burning straw in the field must be reduced or prohibited; although this can improve the nutrition level of soil, the increasing the TOC content will enhance the mercury content, which will then harm human health through the food chain. Moreover, we must reduce the cultivation period in the agricultural land near the woodland in order to reduce the mercury pollution of the woodland.

## Figures and Tables

**Table 1:** The information on the sampling site.

Sampling Site	Sampling Number	Land Use Type	Main Plant	Longitude	Latitude	Interval (cm)	Depth (cm)	Visible Characteristic of Soil
TS1	22	Agricultural land	Rice	101°58′34″	14°56′32″	10	0–210	Gray, clay
TS2	10	Woodland	Oak	104°56′14″	14°33′05″	5	0–18	Humus layer
							18–45	Brown, red, sand
TS3	20	Abandoned agricultural land	Once planted rice	105°10′48″	15°09′00″	5	0–12	Black, humus layer, a few roots
							12–47	Pale yellow
							47–100	Pale yellow, iron rust
							>100	Fe-Mn nodule
TS4	97	Abandoned agricultural land	Once planted rice	104°55′12″	15°04′12″	2	0–20	Gray-black, humus layer
							20–30	Gray, silty sand
							30–40	White, silty sand
							40–65	Yellow soil, silty sand
							65–100	Yellow, a little iron rust
							100–112	Yellow, much Fe-Mn nodule
TS5	81	Agricultural land	Rice	104°24′00″	14°58′48″	5	0–80	Pale yellow, a little roots
							80–105	Gray, iron rust
							105–160	Gray-green, a little Fe-Mn nodule
							160–205	Fe-Mn nodule
							205–290	Gray-green, clay
TS6	10	Woodland	Teak (*Tetona grandis*), ceiba (*Bombax anceps*)	104°30′36″	15°11′59″	5	0–45	A thick fallen leaves layer above the soil

**Table 2:** The geoaccumulation index range and corresponding contamination level.

I_geo_	Level Risk	Contamination Level
<0	Uncontaminated	0
0–1	Uncontaminated/moderately contaminated	1
1–2	Moderately contaminated	2
2–3	Moderately/strongly contaminated	3
3–4	Strongly contaminated	4
4–5	Strongly/extremely contaminated	5
5–6	Extremely contaminated	6

**Table 3:** The content of total mercury (THg) content (μg∙kg^−1^).

	TS1	TS2	TS3	TS4	TS5	TS6
Sampling numbers	22	10	20	59	59	10
Minimum	8.6	15.2	1.3	ND *	ND *	8
Maximum	23.4	26.5	69.4	9.1	40.2	19.1
Mean value	13.25	20.55	8.1	4.19	13.44	13.41
Standard deviation	3.88	2.99	16.01	1.81	10.62	4.11

* No detection, the detection limit is 0.10 μg∙kg^−1^.

**Table 4:** Statistics of the data for the TOC ^a^ (g/kg), Fe (mg/kg), and pH (in unit).

	*n*	Min.	Max.	Mean	SD
TS1					
TOC	22	4.13	15.45	5.90	2.57
Fe	22	43.59	99.21	84.81	11.94
pH	22	5.86	6.78	6.34	0.22
TS2					
TOC	10	4.16	18.83	7.66	4.48
Fe	5	68.92	106.97	82.33	15.28
pH	10	4.93	6.12	5.49	0.47
TS3					
TOC	20	0.40	5.62	1.13	1.32
Fe	10	1.52	66.44	11.12	19.89
pH	20	4.66	5.86	5.51	0.29
TS4					
TOC	59	0.42	7.45	1.58	1.20
Fe	20	5.13	150.76	26.68	31.74
pH	59	5.44	6.72	6.12	0.35
TS5					
TOC	59	0.34	2.78	1.26	0.55
Fe	30	2.79	289.18	59.14	83.11
pH	59	4.40	8.62	6.58	1.39
TS6					
TOC	10	0.94	11.92	2.76	3.35
Fe	6	1.90	7.49	3.02	2.20
pH	10	4.92	5.26	5.10	0.14

^a^ TOC data are from a study by Zhou et al. [44].

**Table 5:** The soil texture in each profile.

Sampling Sites	Land Use Type	Depth (cm)	Clay	Silt	Sand	Soil Texture
			%			
TS1	Agricultural land	0–210	13.6	68.7	17.7	Silty loam
TS2	Woodland	0–45	13.4	49.4	37.2	Silty loam
TS3	Abandoned agricultural land	0–100	3.0	7.9	89.1	Loamy sand
TS4	Abandoned agricultural land	0–60	3.9	18.4	77.7	Sandy loam
		60–100	5.0	40.4	54.7	Loam
TS5	Agricultural land	0–80	5.7	27.0	67.3	Sandy loam
		80–160	6.9	52.5	40.7	Silty loam
		160–200	10.7	41.7	47.6	Loam
		200–290	10.7	71.0	18.3	Silty loam
TS6	Woodland	0–45	4.2	14.0	81.8	Sandy loam

**Table 6:** The ratio of mercury contamination level.

Sampling Sites	Land Use Type	*n*	Contamination Level		
			0	1	2
			%		
TS1	Agricultural land	22	90.91	9.09	0
TS2	Woodland	10	0	100	0
TS3	Abandoned Agricultural land	20	85	10	5
TS4	Abandoned Agricultural land	59	100	0	0
TS5	Agricultural land	59	72.88	23.73	3.39
TS6	Woodland	10	70	30	0
